# A Mixture of *Lactobacillus* HY7601 and KY1032 Regulates Energy Metabolism in Adipose Tissue and Improves Cholesterol Disposal in High-Fat-Diet-Fed Mice

**DOI:** 10.3390/nu16152570

**Published:** 2024-08-05

**Authors:** Kippeum Lee, Hyeon-Ji Kim, Joo-Yun Kim, Jae-Jung Shim, Jae-Hwan Lee

**Affiliations:** R&BD Center, Hy Co., Ltd., 22 Giheungdanji-ro 24 Beon-gil, Giheung-gu, Yongin-si 17086, Republic of Korea; joy4917@hanmail.net (K.L.); hyeonjk@hy.co.kr (H.-J.K.); jjshim@hy.co.kr (J.-J.S.); jaehwan@hy.co.kr (J.-H.L.)

**Keywords:** cholesterol disposal, energy metabolism, thermogenesis, obesity, *Lactobacillus* HY7601 and KY1032

## Abstract

We aimed to characterize the anti-obesity and anti-atherosclerosis effects of *Lactobacillus curvatus* HY7601 and *Lactobacillus plantarum* KY1032 using high-fat diet (HFD)-fed obese C57BL/6 mice. We divided the mice into control (CON), HFD, HFD with 10^8^ CFU/kg/day probiotics (HFD + KL, HY7301:KY1032 = 1:1), and HFD with 10^9^ CFU/kg/day probiotics (HFD + KH, HY7301:KY1032 = 1:1) groups and fed/treated them during 7 weeks. The body mass, brown adipose tissue (BAT), inguinal white adipose tissue (iWAT), and epididymal white adipose tissue (eWAT) masses and the total cholesterol and triglyceride concentrations were remarkably lower in probiotic-treated groups than in the HFD group in a dose-dependent manner. In addition, the expression of uncoupling protein 1 in the BAT, iWAT, and eWAT was significantly higher in probiotic-treated HFD mice than in the HFD mice, as demonstrated by immunofluorescence staining and Western blotting. We also measured the expression of cholesterol transport genes in the liver and jejunum and found that the expression of those encoding liver-X-receptor α, ATP-binding cassette transporters G5 and G8, and cholesterol 7α-hydroxylase were significantly higher in the HFD + KH mice than in the HFD mice. Thus, a *Lactobacillus* HY7601 and KY1032 mixture with 10^9^ CFU/kg/day concentration can assist with body weight regulation through the management of lipid metabolism and thermogenesis.

## 1. Introduction

Overweight and obesity are closely associated with a variety of metabolic disorders, such as type 2 diabetes, dyslipidemia, a range of cancers, hypertension, and cardiovascular disease [1,2]. Obesity is caused by an energy imbalance between calorie intake and consumption [3]; therefore, numerous attempts have been made to combat obesity by preventing excessive energy storage in adipose tissue [4]. Conventionally, adipose tissue is categorized as white adipose tissue (WAT) or brown adipose tissue (BAT) [5,6]. Excess consumption of energy results in the storage of lipids as unilocular fat droplets within WAT adipocytes. In contrast, BAT is composed of cells including multilocular fat droplets and a relatively large number of mitochondria, and these are capable of dissipating this accumulated energy as heat [7,8]. It is known that BAT plays a vital role in the maintenance of body temperature during infancy, but it becomes inactive in adult humans [9]. This unique function of BAT has led to it being regarded as a promising target to treat or prevent obesity [10,11].

Energy metabolism is highly regulated to preserve homeostasis [12]. To date, whereas there have been numerous studies of the systems that regulate food intake, there have been fewer regarding the means of increasing energy dissipation [13]. However, methods for increasing energy consumption are attracting attention because of the challenge of reducing obesity through the management of food intake [14,15]. It is known that energy consumption can be significantly modified by sympathetic signals to peripheral tissues, including BAT and skeletal muscle [16]. In particular, uncoupling protein 1 (UCP1) is greatly expressed in BAT, where it is responsible for proton leakage, which uncouples oxidative phosphorylation, thereby causing heat dissipation [17,18,19]. As interest in the function of BAT and beige adipocytes, which function similarly to BAT adipocytes and have been identified in the WAT of adults, has increased, methods that could increase the activity of these cells have been sought [11]. The stimulation of “browning” in WAT would increase energy consumption, which makes it a potential target for obesity therapeutics [20,21].

Cholesterol is a necessary component of cell membranes and a precursor of crucial steroid hormones and bile acids [22,23,24]. However, a high-fat diet (HFD) reduces intracellular cholesterol concentrations by reducing its uptake and increasing its export [25]. In addition, excess cholesterol in the circulation can lead to hyperlipidemia, cardiovascular disease, and premature death [26]. Liver X receptors (LXRs) are sterol sensors that detoxify and eliminate cholesterol by increasing the reverse transport and catabolism of cholesterol. The expression of LXR in the liver and intestines increases with excess cholesterol and fatty acid consumption [27,28]. The activation of this receptor increases the expression of cholesterol 7a-hydroxylase (CYP7a1), which is responsible for converting cholesterol to bile acids and increases beta-oxidation, which is involved in thermogenesis [29,30]. Furthermore, recent studies have shown that LXRs regulate energy expenditure by regulating BAT activity [31,32].

Recently, various dietary compounds, plant extracts, and lactobacilli have been evaluated as potential new treatments for obesity through their thermogenic effects. Adipocyte browning is considered to be one of the most important mechanisms underlying the anti-obesity effects of extracts, such as caffeine, green tea, flavonoids, *Garcinia cambogia*, and naringin. These natural thermogenic supplements have been shown to increase the resting metabolic rate and therefore overall energy expenditure. Furthermore, thermogenesis was also recently shown to be generated by a probiotic-containing diet. These effects would be expected to accelerate weight loss and the reduction in WAT mass. *Lactobacillus fermentum*, *Lacticaseibacillus paracasei*, *Bifidobacterium longum*, and *Lactobacillus rhamnosus* are reported to reduce obesity by reducing adipogenesis and increasing thermogenesis [33,34,35].

In previous studies, we have demonstrated an inhibitory effect of intake of *Lactobacillus curvatus* HY7601 and *Lactobacillus plantarum* KY1032 on triglyceride accumulation in human adipose [36]. In addition, an antiadipogenic effect of these probiotics has been demonstrated in HFD-fed obese mice, which is associated with reductions in the serum total cholesterol concentration and the adipocyte volumes in epididymal fat [37]. However, the thermogenic effects of the administration of a mixture of *Lactobacillus* HY7601 and KY1032 in the BAT and WAT of HFD-fed mice have not been evaluated. Thus, the aim of the present study was to investigate whether a mixture of *Lactobacillus* HY7601 and KY1032 would cause the browning of WAT and activate the cholesterol circulation system. The data suggest that probiotic mixtures may help prevent obesity by causing the dissipation of energy as heat in adipose tissue as well as the inhibition of fat accumulation that has previously been reported.

## 2. Materials and Methods

### 2.1. Culture of Bacteria and Preparation

*Lactiplantibacillus plantarum* KY1032 (KY1032) and *Lactobacillus curvatus* HY7601 (HY7601) were isolated from kimchi and stocked in a seed culture library at hy Co., Ltd. (Yongin-si, Republic of Korea). KY1032 and HY7601 were cultured anaerobically in de Man, Rogosa, and Sharp (MRS) broth at 37 °C for 24 h. Then, the cultured cells were centrifuged at 8000× *g* at 4 °C for 10 min and washed using phosphate-buffered saline. And, the pellets of cells were resuspended in saline and lyophilized for in vivo experiments.

After freeze-drying, the number of viable cells was measured using the plate counting method. Briefly, 1 g of each LAB freeze-dried powder was suspended in 10 mL of physiological saline and serially diluted. Afterwards, 1 mL of the diluted solution was spread on an MRS (de Man, Rogosa, and Sharpe) agar plate and cultured at 37 °C for 24 h. Colonies were counted to determine the number of viable cells per gram of freeze-dried powder.

### 2.2. Animal Study Design

Six-week-old male C57BL/6 mice were obtained from Doo Yeol Biotech (Seoul, Republic of Korea). After 7 days of acclimatization to a commercial diet (AIN-93G diet), the mice were randomly divided into four groups (*n* = 12 each group): a normal-diet group (CON, AIN-93G diet), an HFD group (60% of energy as fat), an HFD plus low-dose (10^8^ CFU/kg/day, HY7601:KY1032 = 1:1) KY1032 and HY7601 mixture (HFD + KL) group, and an HFD plus high-dose (10^9^ CFU/kg/day, HY7601:KY1032 = 1:1) KY1032 and HY7601 mixture (HFD + KH) group. For in vivo assessments, the mice were housed at 22 ± 1 °C and 55% ± 10% humidity under a 12 h light/dark cycle. The mice were orally administered 100 μL of the KY1032 + HY7601 mixture or equal volumes of saline daily (CON and HFD groups) via oral gavage for 7 weeks. The body weight and food and water intake of all mice were measured every week.

At the end of the experimental period, the mice were sacrificed using carbon dioxide, and samples of blood, feces, epididymal WAT, inguinal WAT, liver, BAT, and spleen were collected. The blood samples were centrifuged at 3000× *g* for 20 min to separate the serum, which was subjected to biochemical testing. The weights of tissue and fecal samples were detected and stored at −80 °C until subsequent evaluation. The animal study was approved by the Institutional Animal Care and Use Committee of hy Co., Ltd. (IACUC, Yongin-si, Republic of Korea; IACUC approval number AEC-2024-0004-Y).

### 2.3. Measurement of Body Temperature

After 7 weeks of treatment, we measured the mice’s rectal temperatures four times by using a thermometer (Testo 925 Type, Testo, Lenzkirch, Germany) and calculated the mean values.

### 2.4. Histologic Analysis

The epididymal WAT, inguinal WAT, and BAT were fixed with 10% (*v/v*) formalin solution (Sigma-Aldrich, St. Louis, MO, USA), and embedded in paraffin. Tissue samples were cut, sectioned, and stained using hematoxylin and eosin (H&E). Section images were obtained using an Olympus CX33 microscope (Olympus, Tokyo, Japan).

Immunofluorescence (IF) staining of the inguinal WAT and BAT sections was performed with anti-UCP-1 polyclonal antibody (diluted 1:250; BS-1925R, Bioss, Woburn, MA, USA), goat anti-rabbit IgG H&L (diluted 1:400, ab150080; Abcam, Cambridge, UK), and 4′-6-diamidino-2-phenylindole (DAPI; Boster, Biological Technology, CA, USA). Stained images were obtained using a ZOE Fluorescent Cell Imager (Bio-Rad Laboratories, Hercules, CA, USA). The histologic analyses were processed by Doo Yeol Biotech (Seoul, Republic of Korea).

### 2.5. Biochemical Analyses

The circulating triglyceride (TG), total cholesterol (T-Chol), high-density lipoprotein cholesterol (HDL-Chol), low-density lipoprotein cholesterol (LDL-Chol), glucose, lactate, blood urea nitrogen (BUN), creatine concentrations, creatine kinase (CK), alanine transaminase (ALT), aspartate transaminase (AST), lactate dehydrogenase (LDH) activities, and HbA1c level were measured at Doo Yeol Biotech (Seoul, Republic of Korea).

### 2.6. Western Blot

Adipose tissues including BAT, epididymal WAT, and inguinal WAT were lysed by using PRO-PREP^TM^ (iNtRON Biotechnology, Seoul, Republic of Korea) buffer supplemented with the Halt protease inhibitor cocktail (Thermo Scientific, Waltham, MA, USA, #78410). Then, the protein concentrations of the lysates were quantified using the DC Bio-Rad Protein Assay (Bio-Rad Laboratories, Hercules, CA, USA), according to the commercial protocol. For Western blotting, 18ug proteins were diluted with 5 × sample buffer (50 mM Tris at pH 6.8, 2% SDS, 10% glycerol, 5% β-mercaptoethanol, and 0.1% bromophenol blue) and boiled for 3 min at 95 °C before performing SDS-PAGE (sodium dodecyl sulfate–polyacrylamide gel electrophoresis). The constituent proteins were resolved in sodium dodecyl sulfate–polyacrylamide gel electrophoresis and transferred to polyvinylidene fluoride membranes. The membranes were blocked in 5% skim milk, rinsed with Tris-buffered saline buffer, including Tween 20 (Sigma-Aldrich), and then incubated with primary antibody overnight at 4 °C. The membranes were incubated with secondary antibody conjugated to IgG horseradish peroxidase. The antibodies used were obtained from Cell Signaling Technology (Danvers, MA, USA), and they are listed in Table 1. Reactive bands were visualized using a chemiluminescence system with LAS image software (ImageQuant^TM^ LAS 4000, version 1.2, Build 1.2.1.119, Fuji, New York, NY, USA).

### 2.7. RNA Extraction and Gene Expression Analysis

RNA was isolated from adipose and liver tissue samples using an Easy-spin Total RNA Extraction Kit (iNtRON Biotechnology), according to manufacturer’s protocol. The extracted RNA was reverse-transcribed to cDNA using a Maxime RT PreMix (LiliF^TM^ Diagnostics, Seoul, Republic of Korea). Then, 1 μg of RNA with an A260/280 ratio between 1.90 and 2.10 was used for cDNA synthesis. cDNA was amplified with a QuantStudio 6-Flex Real-time PCR System (Applied Biosystems, Fosster City, CA, USA), Gene Expression Master Mix (Applied Biosystems), and Taqman assays (Applied Biosystems). Target genes and the Taqman probes used in the real-time PCR are listed in Table 2. Amplifications were performed with the following thermal cycling conditions: initial activation of 10 min at 95 °C, followed by 50 cycles of 30 s of denaturation at 95 °C, 30s of annealing at 54–58 °C, and 30 s of elongation at 72 °C. The expression of the genes was normalized to that of *Gapdh*.

### 2.8. Fecal Cholesterol and Bile Acid Analysis

To obtain cholesterol and bile acid, the stool samples were extracted with 200 μL of a chloroform: isopropanol: NP-40 (7:11:0.1) mixture in a micro-homogenizer. The extracts were centrifuged at 15,000× *g* for 10 min, and the liquids (organic phase) were transferred to new tubes. All samples were dried at 50 °C to remove chloroform. The fecal cholesterol and bile acid concentrations of the mice were evaluated using a Total Cholesterol Assay Kit (BM-CHO-100, Biomax, Guri-si, Gyeonggi-do, Republic of Korea) and a Bile Acid Assay Kit (Colorimetric; ab239702, Abcam), respectively, according to the manufacturers’ protocols.

### 2.9. Statistical Analysis

All data are demonstrated as the mean ± standard deviation (SD) and were statically analyzed through one-way ANOVA, followed by Duncan’s test. The analyses were performed using SPSS version 26.0 (IBM, Inc., Armonk, NY, USA). *p* < 0.05 was considered significant (a > b > c > d).

## 3. Results

### 3.1. A Lactobacillus HY7601 and KY1032 Mixture Ameliorates HFD-Induced Obesity

**Figure 1:**
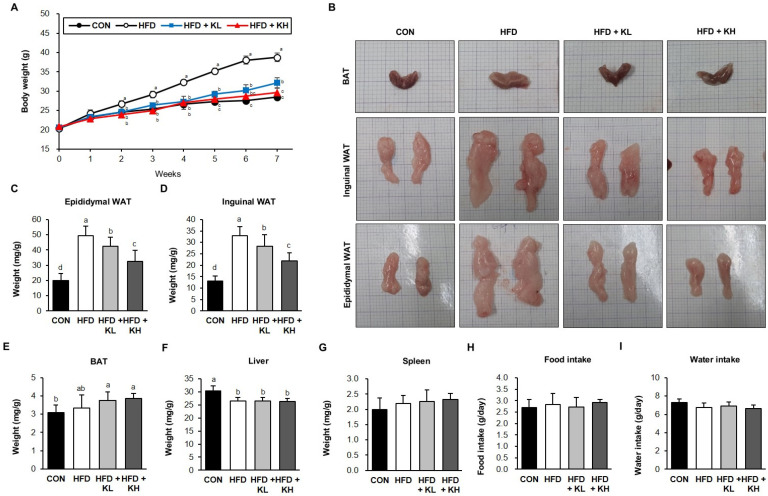
Effects of *Lactobacillus* HY7601 and KY1032 on the body and tissue masses of the mice. (**A**) Body masses of the mice after 7 weeks. (**B**) Images of brown adipose tissue (BAT), inguinal white adipose tissue (inguinal WAT), and epididymal adipose tissue (epididymal WAT) samples. Masses of the (**C**) epididymal WAT, (**D**) inguinal WAT, (**E**) BAT, (**F**) livers, and (**G**) spleens of the mice. (**H**) Food intake and (**I**) water intake per unit of body weight. Data are expressed as mean ± SD (n = 12). Groups accompanied by different letters were significantly different: *p* < 0.05 (a > ab > b > bc > c > d). CON, control; HFD, HFD-fed obese mice; HFD-KL, 10^8^ CFU/kg/day *Lactobacillus* HY7601 and KY103 plus HFD; HFD-KH, 10^9^ CFU/kg/day *Lactobacillus* HY7601 and KY103 plus HFD.

To examine the effects of the probiotics HY7601 and KY1032 on adiposity, we induced obesity in 6-week-old mice by feeding an HFD for 7 weeks. As shown in Figure 1A, HFD feeding caused larger increases in body mass than a normal diet (CON). The average body mass of the HFD group at week 7 was 38.7 g, but those of the HFD + KL and HFD + KH groups, which were administered the *Lactobacillus* mixture along with the HFD, were 32.1 g and 29.6 g, respectively. As shown in Figure 1B–F, the mice in the HFD + KL and HFD + KH groups had 249% and 252% heavier eWAT and iWAT depots, respectively, than the CON mice. However, the HFD + KL mice had less eWAT (85.8%) and iWAT (86.1%) than the HFD mice. The HFD + KH mice had lower eWAT (66.0%) and iWAT (66.7%) masses than the HFD mice. However, there were no significant differences in the masses of the BAT or spleen between the groups (Figure 1F,H). The masses of the livers, normalized to the body masses of the three HFD-fed mice, were significantly lower than those of the CON mice, owing to their larger adipose depots. Finally, there were no differences in the food or water consumption of the four groups, which implies the differences in body weight were not the result of differences in dietary intake (Figure 1H–I). These findings indicate that HFD-induced obesity in mice can be ameliorated through dietary supplementation with a *Lactobacillus* HY7601 and KY1032 mixture.

### 3.2. The Lactobacillus HY7601 and KY1032 Mixture Reduces the Expansion of Adipose Tissue in HFD-Fed Mice

**Figure 2:**
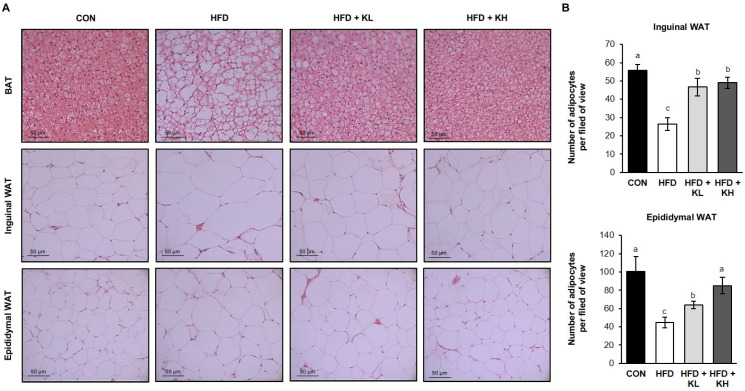
Histology of adipose tissue depots in each of the mouse groups. (**A**) Brown adipose tissue (BAT, top), inguinal white adipose tissue (WAT, middle), and epididymal white adipose tissue (bottom), stained with hematoxylin and eosin (scale bar = 50 μm). (**B**) Number of adipocytes in epididymal WAT and inguinal WAT, determined using ImageJ (version 1.53t). N = 6–8 mice/group. Data are expressed as mean ± SD (n = 12). Groups accompanied by different letters were significantly different: *p* < 0.05 (a > b > c).

Excessive calories of an HFD cause the expansion of adiposity for the storage of neutral lipids, mediated by increases in the number and proportion of adipocytes [38]. As shown in Figure 2B, significantly larger adipocytes were identified in the eWAT and iWAT of HFD mice than in the equivalent depots in CON mice. We also found that supplementation with the *Lactobacillus* mixture was associated with smaller eWAT and iWAT depots. The mean number of adipocytes per image area in the iWAT was 55.6 ± 5.1 in the CON mice but only 26.3 ± 3.5 in the HFD mice due to increased adipocytes size. However, both doses of the probiotics HY7601 and KY1032 normalized the numbers because they reduced the adipocyte size. Similarly, the mean number of adipocytes in the eWAT was 100.3 ± 16.6 per unit area in the CON group but only 44.6 ± 5.7 per unit area in the HFD group, and those of the HFD-fed mice were increased by the probiotics in a concentration-dependent manner. This increase in the number of adipocytes per unit area in the probiotic-fed groups means that the size of lipid droplets is significantly reduced. Interestingly, the BAT masses did not significantly differ between the four groups, although the brown adipocytes differed in size between the CON and HFD groups when sections were examined histologically (Figure 2).

### 3.3. The Lactobacillus HY7601 and KY1032 Mixture Affects the Serum Lipid Profile of the Mice

**Figure 3:**
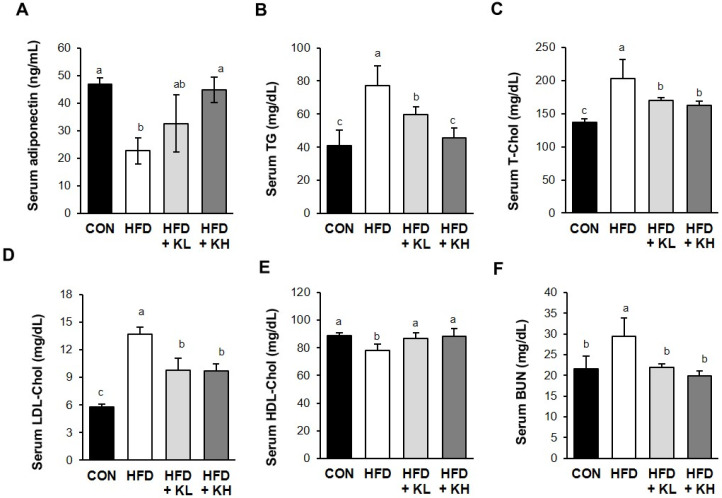
Effects of *Lactobacillus* HY7601 and KY1032 on serum lipid and cholesterol-related parameters in HFD-fed mice. Serum concentrations of (**A**) adiponectin, (**B**) triglyceride (TG), (**C**) total cholesterol (T-Chol), (**D**) low-density lipoprotein cholesterol (LDL-Chol), (**E**) high-density lipoprotein cholesterol (HDL-Chol), and (**F**) blood urea nitrogen (BUN). Data are mean ± SD (n = 12). Groups accompanied by different letters were significantly different: *p* < 0.05 (a > ab > b > c).

The serum adiponectin and lipid-related parameters were measured next. *Lactobacillus* HY7601 and KY1032 increased the serum adiponectin concentrations when administered at 10^8^ CFU/kg/day or 10^9^ CFU/kg/day by 32.6 ± 6.3 pg/mL and 44.8 ± 10.0 pg/mL, respectively, compared with the HFD mice (Figure 3A). Additionally, the serum TG, T-Chol, and LDL-Chol concentrations of the CON group were 40.8 mg/dL, 136.8 mg/dL, and 5.8 mg/dL, while those of the HFD group were significantly higher at 77.2 mg/dL, 203.2 mg/dL, and 13.7 mg/dL, respectively. However, the serum TG, T-Chol, and LDL-Chol concentrations of the HFD group administered the *Lactobacillus* mixture were significantly reduced by both doses (Figure 3B–D). However, the HFD + KL group had lower serum TG (77.3%), T-Chol (83.7), and LDL-Chol (71.4%) concentrations than the HFD group. The HFD + KH group had lower TG (59.1%), T-Chol (79.9%), and LDL-Chol (70.6%) concentrations than the HFD group. However, both probiotic treatments returned the HDL-Chol concentrations (111.1% and 112.9%) to those of the CON mice, such that it significantly differed from that of the HFD group, as shown in Figure 3E. Finally, the BUN was 136.7% higher in the HFD group than in the CON group, consistent with a higher risk of obesity and diabetes, but it was normalized by both doses of probiotics (Figure 3F).

### 3.4. The Lactobacillus HY7601 and KY1032 Mixture Affects the Energy Metabolism of the Mice

**Figure 4:**
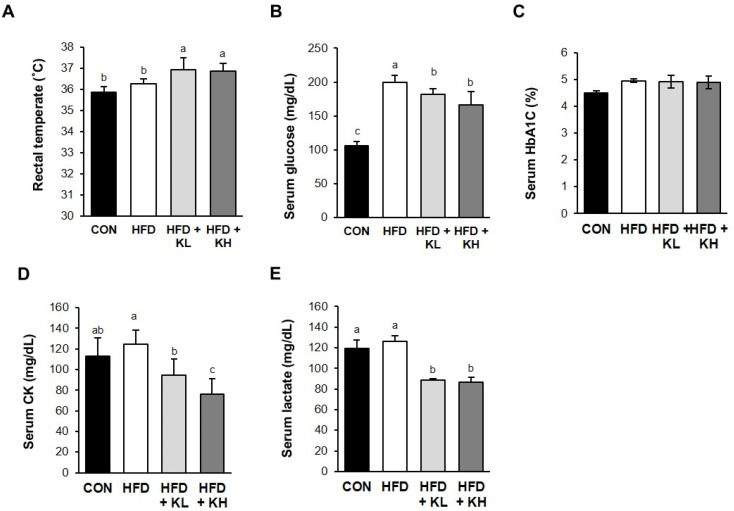
Effects of *Lactobacillus* HY7601 and KY1032 on the rectal temperature and serum parameters related to glucose metabolism in HFD-fed mice. (**A**) Core temperature, measured using a thermometer. Serum concentrations of (**B**) glucose, (**C**) glycated or glycosylated hemoglobin A1c (HbA1C), (**D**) creatine kinase (CK), and (**E**) lactate. Data are expressed as mean ± SD (n = 12). Groups accompanied by different letters were significantly different: *p* < 0.05 (a > ab > b > c).

To determine whether probiotics HY7601 and KY1032 have a thermogenic effect in HFD-fed obese mice, we measured their body temperatures using a rectal temperature. We found that the probiotics significantly increased the body temperatures of the mice compared to both the HFD-fed and CON mice, as shown in Figure 4A. A recent study reported that BAT is specialized in the heat production caused by cold- or diet-induced thermogenic effects. Because it metabolizes the production of heat by burning fat and controls glucose homeostasis and insulin levels, this fat is considered an effective target for anti-obesity treatment [39]. Therefore, body temperature is frequently measured to identify heat generation by mice in studies of the effects of food supplements [40]. In addition, because obesity has deleterious effects on plasma-free fatty acid concentrations and glucose homeostasis, we also determined the effects of the probiotics HY7601 and KY1032 on these parameters. The serum fasting blood glucose concentrations of the HFD group were 190% higher than those of the CON group, and probiotic administration reduced this in a dose-dependent manner to 90.8% and 83.3% (HFD + KL and HFD + KH, respectively) of the CON values (Figure 4B). HFD intake significantly increased the serum HbA1c level compared with the CON mice, but the mixture of *Lactobacillus* HY7601 and KY1032 did not affect this (Figure 4C). Also, the HFD + KL and HFD + KG mice had lower serum CK (75.9%) and (61.0%) concentration than the HFD mice (Figure 4D). The circulating lactate concentration is known to cause induction in the presence of hyperinsulinemia and in the early stages of diabetes [41]. The serum lactate concentrations of the mice were not affected by HFD feeding, but the probiotic mixture reduced this (Figure 4E). 

### 3.5. The Lactobacillus HY7601 and KY1032 Mixture Increases UCP1 Activation in Adipose Tissue

**Figure 5:**
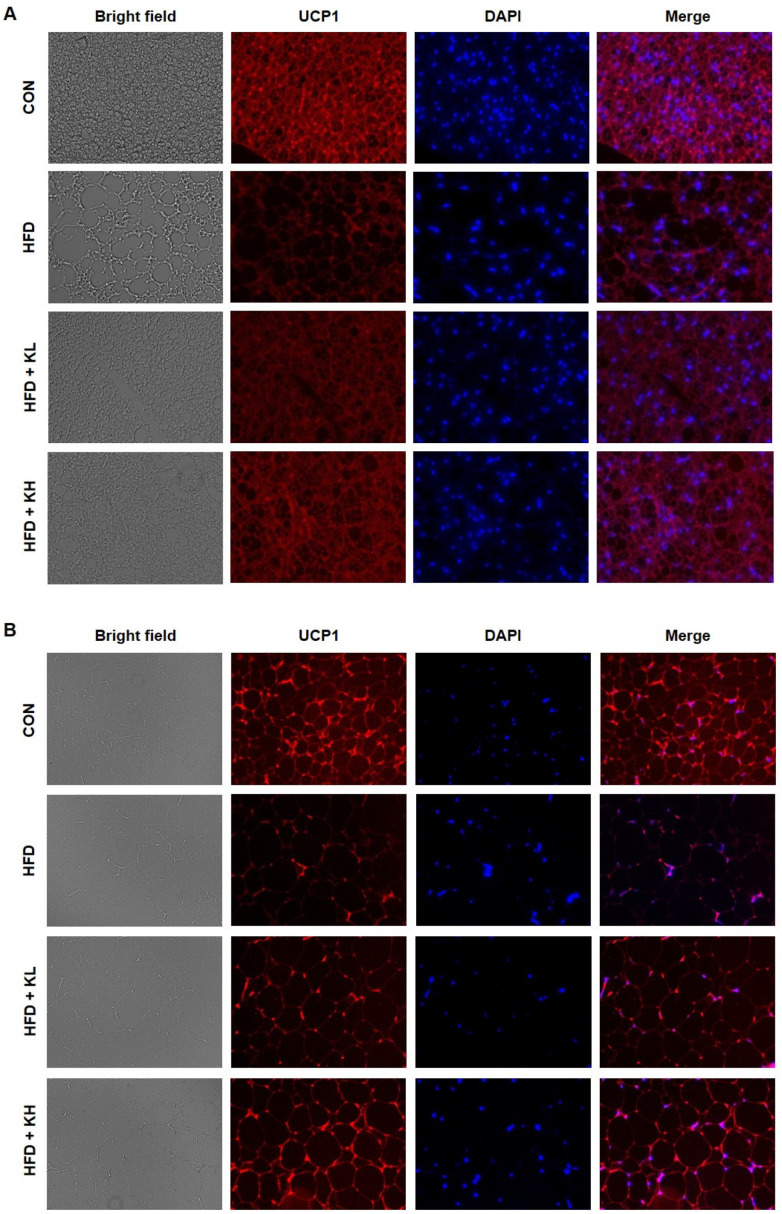
Adipose sections immunostained for uncoupling protein 1 (UCP1) in the various mouse groups. Representative images of (**A**) BAT and (**B**) inguinal WAT (bright field, UCP1 (red), DAPI (blue), and merged UCP1 and DAPI).

We next compared the expression of UCP1, an important mediator of thermogenesis, in the BAT and a representative subcutaneous WAT depot of the various mouse groups using fluorescence staining. As shown in Figure 5, this revealed that the adipose depots of the HFD-fed mice had lower UCP1 expression than those of the CON mice. However, the fluorescence staining intensity corresponding to UCP1 was dose-dependently increased by the probiotic administration in both the BAT and subcutaneous WAT. These results indicate that the probiotic mixture promotes browning and therefore would increase energy dissipation as heat.

### 3.6. The Lactobacillus HY7601 and KY1032 Mixture Increases the Expression of Thermogenic Factors in Adipose Tissue

**Figure 6:**
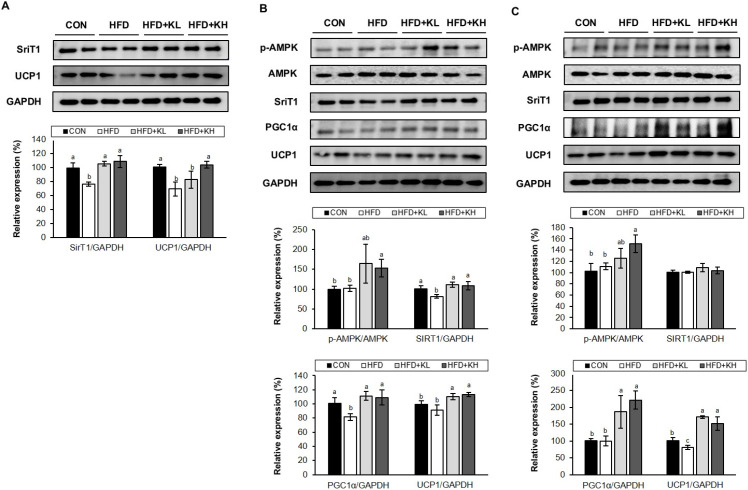
Effects of *Lactobacillus* HY7601 and KY1032 on the expression of key thermogenic proteins in (**A**) BAT, (**B**) inguinal WAT, and (**C**) epididymal WAT. Western blot data for sirtuin 1 (SirT1), UCP1, phosphorylated-AMP-activated protein kinase (*p*-AMPK), AMPK, peroxisome proliferator-activated receptor gamma coactivator 1-alpha (PGC1α), and glyceraldehyde 3-phosphate dehydrogenase (GAPDH). Groups accompanied by different letters were significantly different: *p* < 0.05 (a > ab > b > c).

To further characterize the effects of *Lactobacillus* HY7601 and KY1032 on thermogenesis, we evaluated the expression of proteins involved in the mice’s BAT. UCP1 is a mitochondrial uncoupling protein that is expressed at high levels in BAT, and it stimulates energy expenditure, which can reduce body mass [42]. SirT1 is an NAD-dependent protein deacetylase that induces the browning of WAT [43]. The SirT1 and UCP1 activation in the BAT of the HFD-fed mice was 76.7% and 59.6%, respectively, lower than that in the CON mice, but it was significantly accumulated through treatment with the probiotics at a high dose (Figure 6A). Interestingly, the expression ratio of phosphorylated AMP-activated protein kinase (*p*-AMPK)/AMPK in the inguinal and epididymal WAT of the CON mice did not significantly differ from that in the equivalent depot of the HFD mice. However, the higher dose of probiotics significantly increased it up to 153.1% and 151.2%, respectively. In addition, the levels of SirT1, PGC1α, and UCP1 expression in the inguinal WAT of the HFD were significantly lower (81.5%, 81.5% and 91.2%, respectively) than those of the CON group, but *Lactobacillus* HY7601 and KY1032 administration near-normalized these. In contrast, whereas the PGC1α and UCP1 protein expression levels in the eWAT were lower (99.7% and 80.78%) in the HFD mice than in the CON mice and upregulated by the probiotics, the SriT1 protein level in the eWAT was not significantly affected by HFD or probiotic intake. These findings demonstrate that the *Lactobacillus* mixture increases the protein expression of key thermogenic mediators in BAT and WAT depots, consistent with an increase in non-shivering thermogenesis.

### 3.7. The Lactobacillus HY7601 and KY1032 Mixture Induces Cholesterol Disposal and the Fatty Acids’ β-Oxidation in the Liver

**Figure 7:**
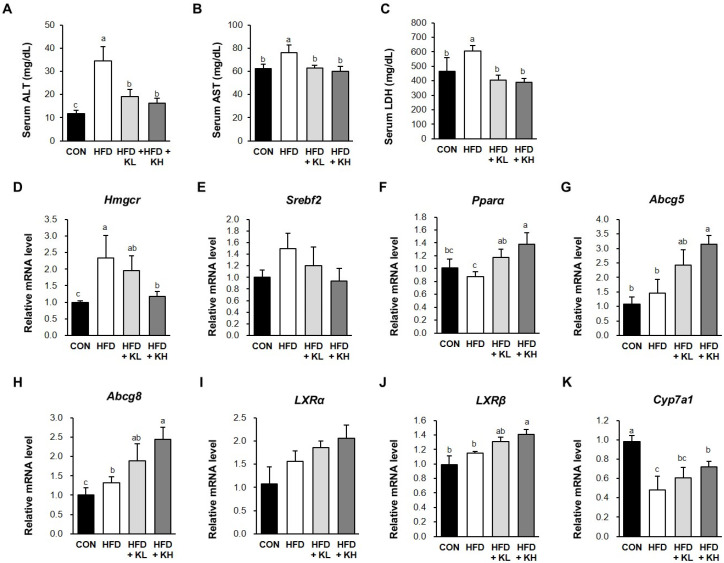
Effects of *Lactobacillus* HY7601 and KY1032 on the circulating concentrations of liver-related enzymes and the liver mRNA expression of the mouse groups. Serum activities of (**A**) alanine transferase (ALT), (**B**) aspartate transaminase (AST), and (**C**) lactate dehydrogenase (LDH). Expression of the genes encoding (**D**) 3-hydroxy-3-methylglutaryl-coenzyme A reductase (*Hmgcr*), (**E**) sterol regulatory element-binding protein 2 (*Srdbp2*), (**F**) peroxisome proliferator-activated receptor alpha (*PPARa*), (**G**) ATP-binding cassette (ABC) transporter G5 (*Abcg5*), (**H**) *Agcg8*, (**I**) liver X receptor alpha (*LXRb*), (**J**) *LXRβ*, and (**K**) cholesterol 7 alpha-hydroxylase (*Cyp7a1*), normalized to that of *Gapdh*. Groups accompanied by different letters were significantly different: *p* < 0.05 (a > ab > b > bc > c).

The serum aspartate aminotransferase (AST), alanine aminotransferase (ALT), and lactate dehydrogenase (LDH) levels of mice are increased by liver disease and obesity [44,45]. Figure 7A–C show that the serum ALT, AST, and LDH activities in the CON mice were 11.8 ± 1.3, 62.1 ± 4.2, and 464.4 ± 95.0, while in the HFD mice they were 34.4 ± 6.3, 76.0 ± 6.6, and 605.5 ± 37.3 respectively. And, these were significantly reduced to 16.1 ± 2.2, 60.0 ± 4.4, and 388.8 ± 27.2 by the higher dose of probiotics. We next evaluated the effects of the *Lactobacillus* mixture on the expression of key mediators of hepatic metabolism (PPARα, Hmgcr, SREBP2, ABCG5, ABCG8, LXRα, LXR, and CYP7A1) in the livers of the mice through qRT-PCR. In particular, we aimed to determine whether the *Lactobacillus* mixture activates cholesterol disposal through increases in LXR expression and the β-oxidation of fatty acids of livers in HFD-fed mice. Figure 7D,E show that the 10^9^ CFU/kg/day dose of the HY7601 and KY1032 probiotic mixture reduced the mRNA expression of *Hmgcr* and *Srebp2* in the HFD mice. Conversely, the expression of the six mediators of liver cholesterol secretion were significantly higher in the HFD-fed mice treated with probiotics than in the HFD-fed control mice (Figure 7F–K). In particular, after treatment with high concentrations of 10^9^ CFU/kg/day, the expression of PPARα (1.56-fold), Abcg5 (2.14-fold), Abcg8 (1.84-fold), LXRα (1.48-fold), LXRβ (1.26-fold), and Cyp7a1 (1.50-fold) was higher than in the HFD-fed mice, although the differences in LXRα expression were not significant according to ANOVA.

### 3.8. The Lactobacillus HY7601 and KY1032 Mixture Stimulates Cholesterol Excretion from the Jejunum

**Figure 8:**
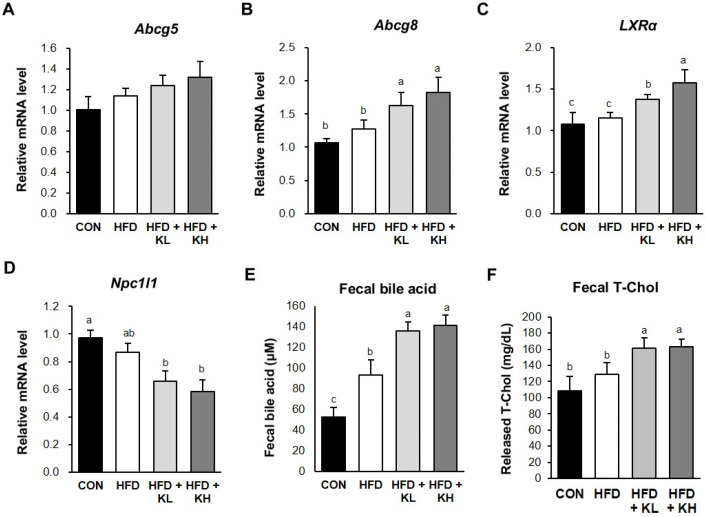
Effects of the *Lactobacillus* HY7601 and KY1032 mixture on cholesterol-metabolism-related parameters in the jejuna of the mouse groups. Expression of genes encoding (**A**) ATP-binding cassette (ABC) transporter G5 (*Abcg5*), (**B**) *Agcg8*, (**C**) liver X receptor alpha (*LXRα*), and (**D**) NPC1-like intracellular cholesterol transporter 1 (*Npcl1*), normalized to that of *Gapdh*. Fecal concentrations of (**E**) bile acids and (**F**) total cholesterol. Groups accompanied by different letters were significantly different: *p* < 0.05 (a > ab > b > c).

Excess cholesterol in the plasma travels to intestinal cells and is excreted by ABCG5 and AGCG8 complexes [46], and the transcription factor LXRα promotes ABCG5/G8 expression, which is necessary for intestinal cholesterol disposal [47]. Furthermore, Niemann–Pick C1-Like 1 (NPC1L1) protein is important for jejunum cholesterol transport [48]. It is reported that *Lactobacillus* strains ameliorate cholesterol levels via the regulation of gene expression involved in the oxidation and metabolism of cholesterol and bile acid excretion [49]. Therefore, in this study, we investigated whether the *Lactobacillus* HY7601 and KY103 mixture has beneficial effects on cholesterol metabolism in HFD-fed mice. As indicated in Figure 8A–D, the gene levels encoding proteins involved in cholesterol transport in the jejunum of the HFD-fed mice were altered by the probiotic mixture. The mRNA level of *Abcg5* did not differ between the HFD-fed groups. The low and high doses of probiotics increased the *Abcg8* mRNA level by 1.28-fold and 1.43-fold, respectively, vs. the HFD mice and increased the *LXRα* gene level by 1.19-fold and 1.37-fold, respectively. Bile acid is one of the cholesterol metabolites that is exclusively produced in the liver, and, as shown in Figure 8E, the fecal bile acid concentrations of the HFD group were increased by the *Lactobacillus* mixture [50]. Finally, the fecal total cholesterol concentrations of the HFD-fed mice were significantly increased by the high dose of probiotics (Figure 8F). The fecal concentrations of bile acids and total cholesterol tended to be dose-dependently increased by the probiotics, but the difference between each group has no significance [51].

## 4. Discussion

Given the increasing global prevalence and health concerns associated with obesity, it is pivotal to identify effective treatment and prevention strategies [52]. Browning, the process whereby WAT develops characteristics of BAT, including thermogenic capability, is a promising therapeutic target for obesity because it induces the loss of body mass and improves blood glucose control [53]. In addition, it is reported that BAT requires fatty acid oxidation to promote heat generation [21]. This process enhances thermogenesis through the transfer of acyl-CoA from the cytoplasm to the mitochondrial matrix, resulting in the fatty acids’ beta-oxidation in mitochondria [54]. Therefore, there has been an increase in studies of natural substances that could prevent obesity by reducing body fat accumulation, promoting beta-oxidation, and increasing thermogenesis by browned WAT [11,55].

Probiotics are living microorganisms that have worthwhile effects on human health, such as anti-inflammatory, anti-diabetic, and anti-hypercholesterolemic effects [56]. *Lactobacillus* strains have traditionally been proven to be safe enough to be used as starters for food preservation, dairy products, and fermented vegetables, and they have been used in various human clinical trials [57]. Of these, *Lactobacillus* strains are known to have potent inhibitory effects on obesity through fat accumulation reduction, and an effect on increasing heat generation through the browning of WAT would be of great interest [58]. Furthermore, *Lactobacillus* strains play a crucial role in the regulation of intestinal cholesterol metabolism and transport [59,60]. Previous studies have illustrated the anti-obesity effects of mixtures of *Lactobacillus curvatus* HY7601 and *Lactobacillus plantarum* KY1032, mediated through the regulation of adipogenic factors, such as adiponectin and leptin, by means of both animal experiments and clinical trials [61]. We also investigated whether HY7601 and KY1032 alter the gut microbiota by increasing beneficial bacteria, such as Bifidobacteriaceae and Akkermansiaceae, and decreasing Prevotellaceae and Selenomonadaceae in humans [61]. Moreover, *L. curvatus* HY7601 and *L. plantarum* KY1032 probiotics have been listed as a new FDA NDI health food ingredient and obtained Self-Affiliated Generally Recognized As Safe (GRAS) recognition. However, the mechanisms whereby *Lactobacillus curvatus* HY7601 and *Lactobacillus plantarum* KY1032 enhance thermogenesis and cause the loss of excess cholesterol in HFD-fed obese mice had not been elucidated. Therefore, we aimed to characterize the effects of *Lactobacillus* HY7601 and KY1032 on thermogenesis and cholesterol fluxes in HFD-fed obese mice in this study.

During the 7 weeks of the experiments, the HFD group showed large increases in body weight and in the masses of the iWAT, eWAT, and BAT depots, which were significantly ameliorated by *Lactobacillus* HY7601 and KY1032 supplementation. H&E staining of the fat depots showed that probiotic administration decreased the size and proportion of the lipid droplets in all three. However, the food and water consumption of the four groups of mice did not differ. In addition, treatment with the *Lactobacillus* mixture did not affect HbA1c, an index of long-term glycemic control, but significantly reduced the high serum fasting blood glucose concentration associated with obesity. Interestingly, although the difference was not significant, the core temperatures of the probiotic-fed groups were slightly higher than those of the CON and HFD mice. These data suggest that this probiotic mixture may cause a promotion in energy consumption in obese mice.

The iWAT and eWAT, which are types of white fat, play important roles in the regulation of energy balance and lipid homeostasis. Both tissues are known to be able to be reassembled to have a similar phenotype to BAT, which are called “beige adipose tissue” [62]. This beige fat has a higher mitochondrial content and high expression of BAT-specific genes, such as PGC1α and UCP1. The expression of UCP1 located in the inner mitochondrial membrane and PGC1α, which improves fatty acid and lipid catabolism, is highly associated with the thermogenic capacity. In particular, it is known that increased UCP1 expression leads to shivering thermogenesis, helping to maintain body temperature [21,63]. In this study, the high body temperature of the *Lactobacillus*-HY7601- and KY1032-treated mice suggested an upregulation of thermogenesis in adipose tissue. Consistent with this, immunofluorescence and Western blotting showed that the probiotics effectively increased the UCP1 expression in the BAT, iWAT, and eWAT. Furthermore, the expression of SIRT1, a key metabolic sensor in adipose tissue, was also significantly increased in these adipose depots by the probiotic treatment. Finally, the expression of Pgc1α was also significantly upregulated by this treatment in the iWAT and eWAT by the probiotic treatment vs. HFD-fed mice. SirT1 and PGC1α promote UCP1 expression in WAT, which can cause the browning of adipocytes to produce beige adipocytes. To sum up, *Lactobacillus* HY7601 and KY1032 could induce differentiation into beige adipocytes in WAT, which was demonstrated by UCP1 activation and rectal temperature.

Obesity is associated with various risk factors for disease, including high serum triglyceride, HDL-Chol, LDL-Chol, and glucose concentrations and high blood pressure [51,64,65]. These abnormalities are features of the metabolic disease and can be related to lower energy consumption, in the form of fatty acid oxidation, in adipose tissue. In addition, metabolic syndrome and dyslipidemia are associated with the development of intravascular glucose concentration or insulin resistance, which may be explained by a promotion in the hepatic fatty acids’ beta-oxidation [66]. In this study, the mice in the HFD group had high serum TG, total-Chol, and LDL-Chol concentrations and low serum HDL-Chol concentrations compared to those of the CON group. Both doses of *Lactobacillus* HY7601 and KY1032 reduced the serum TG, total-Chol, and LDL-Chol concentrations and increased the serum HDL-Chol concentrations of the HFD-fed mice. Moreover, this mixture dose-dependently increased the serum adiponectin concentrations of the HFD-fed mice.

Cholesterol synthesized in liver tissue is transported to the intestines as a precursor of bile acid, where it aids in the digestion of dietary lipid [67]. *Lactobacillus* microorganisms regulate the amount of cholesterol that is converted into bile acids. In addition, conjugated bile acids play a role as an emulsifier to aid in the absorption of dietary fats because of their amphiphilic nature [68]. The mechanism whereby *Lactobacillus* improves energy homeostasis through the regulation of cholesterol circulation has not previously been reported. However, numerous studies have indicated that proteins included in fatty acid oxidation, such as cholesterol 7α-hydroxylase (CYP7A1), are essential for heat generation [68]. Thus, the probiotics may cause thermogenesis through an induction of fatty acid oxidation, which is associated with a reduction in cholesterol concentration. To determine the effects of the *Lactobacillus* HY7601 and KY1032 mixture on cholesterol and bile acid excretion, we detected the fecal concentrations of bile acids and cholesterol of the mice and found that the probiotic mixture significantly elevated the fecal cholesterol and bile acid excretion of the HFD-fed mice. These findings indicate that treatment with this probiotic mixture promotes the disposal of cholesterol and bile acids by obese mice. In addition, we measured the effects of the mixture on the mRNA expression associated with cholesterol metabolism in the livers of the mice and found that the expression of *Abcg5*, *Abcg8*, *Lxra*, and *Lxrb* was higher than that of the HFD mice, but only significantly so when the higher dose was administered. Furthermore, the serum activities of ALT, AST, and LDH were decreased by the *Lactobacillus* mixture in HFD-fed mice. In summary, the *Lactobacillus* HY7601 and KY1032 mixture reduces cholesterol concentrations by inhibiting the reabsorption of cholesterol in the intestinal tissue and increasing the bile acids’ excretion.

## 5. Conclusions

In conclusion, we have shown that a mixture of *Lactobacillus curvatus* HY7601 and *Lactobacillus plantarum* KY1032 improves the energy metabolism and fats status of HFD-fed obese mice. These probiotics significantly enhance thermogenesis by increasing UCP1 expression and causing the browning of WAT, which contributes to their anti-obesity effects. Moreover, they inhibit intestinal and liver cholesterol absorption, further limiting obesity and dyslipidemia. These results imply that probiotics may influence the improvement of lipid metabolic function, but it is not yet known how this effect is associated with gut microbiota change, which will be investigated in future studies. To sum up, our data suggest that a mixture of HY7601 and KY1032 may represent a potential therapeutic strategy for overweight and obesity.

## Figures and Tables

**Table 1:** Antibody list used in Western blot analysis.

Antibody Target	Catalog No.
Glyceraldehyde-3-phosphate dehydrogenase	CS 2188
Uncoupling protein 1	CS 77298
Phosphorylated adenosine monophosphate-activated protein kinase (Thr172)	CS 50081
Adenosine monophosphate-activated protein kinase	CS 2535
Peroxisome proliferator-activated receptor gamma coactivator 1-alpha	CS 2178
Sirtuin-1	CS 2028

**Table 2:** List of the target genes and corresponding Taqman probe kits used in gene expression analysis.

Gene	Product Name	Catalog No.
*Gapdh*	Glyceraldehyde-3-phosphate dehydrogenase	Mm99999915_g1
*Pparα*	Peroxisome proliferator-activated receptor alpha	Mm00440939_m1
*Hmgcr*	3-hydroxy-3-methylglutaryl-coenzyme A reductase	Mm01282499_m1
*Srebf2*	Sterol regulatory element-binding factor 2	Mm01306292_m1
*Abcg5*	ATP-binding cassette, sub-family G (WHITE), member 5	Mm00446241_m1
*Abcg8*	ATP-binding cassette, sub-family G (WHITE), member 8	Mm00445980_m1
*Nr1h3 (Lxrα)*	Nuclear receptor subfamily 1, group H, member 3	Mm00443451_m1
*Nr1h21 (Lxrβ)*	Nuclear receptor subfamily 1, group H, member 2	Mm00437265_g1
*Cyp7a1*	Cytochrome P450, family 7, subfamily a, polypeptide 1	Mm00484150_m1
*Npc1l1*	NPC1-like 1	Mm01191972_m1

## Data Availability

All of the data are contained within the article.
